# Supplementary figures and images for: Modelling and classifying joint trajectories of self-reported mood and pain in a large cohort study
Source: PLOS Digit Health. 2023 Mar 30;2(3):e0000204. doi: 10.1371/journal.pdig.0000204 (PMC10062665; doi:10.1371/journal.pdig.0000204)

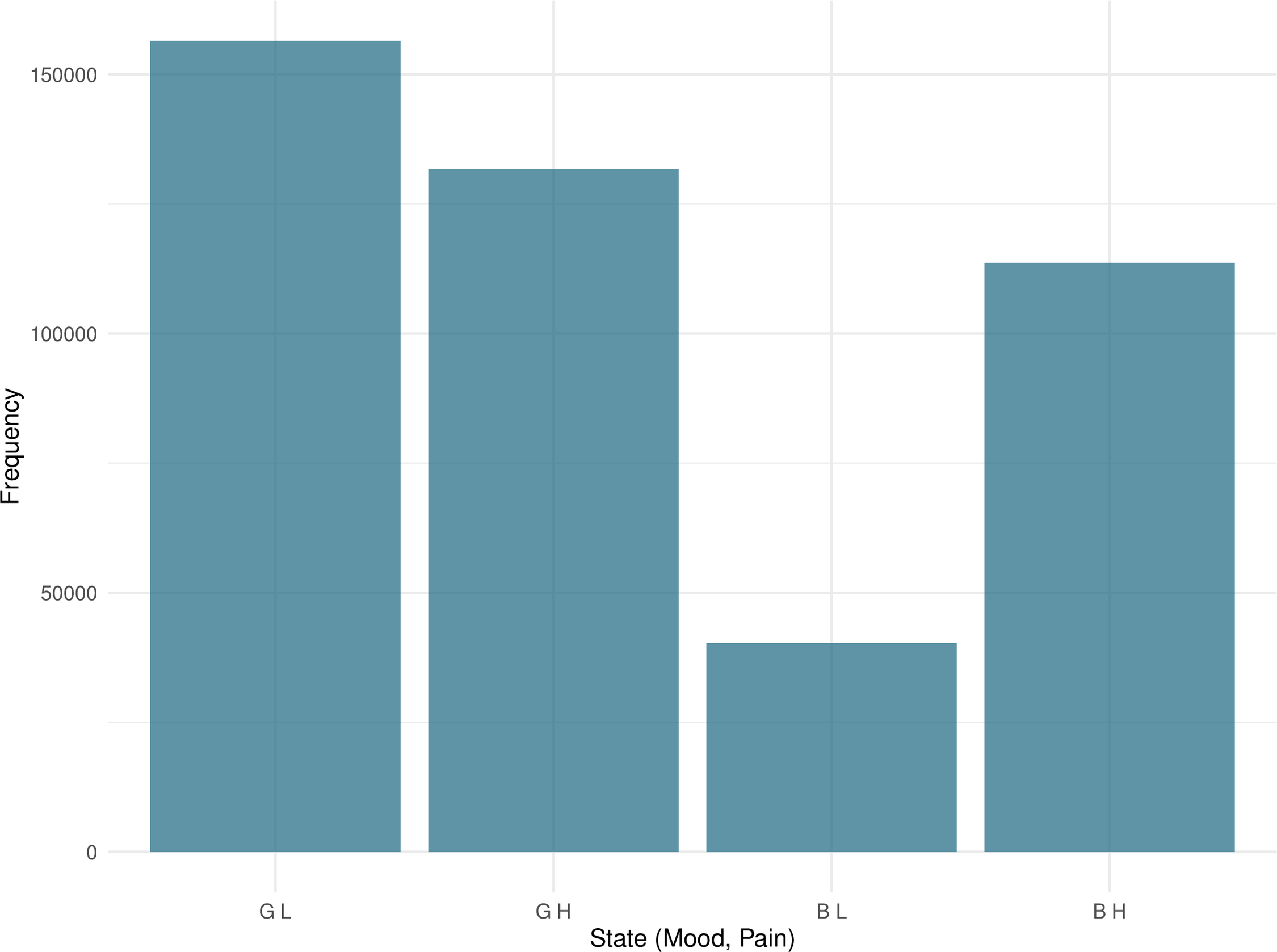

Supplement: S1 Fig — (TIF) [file pdig.0000204.s001.tif]

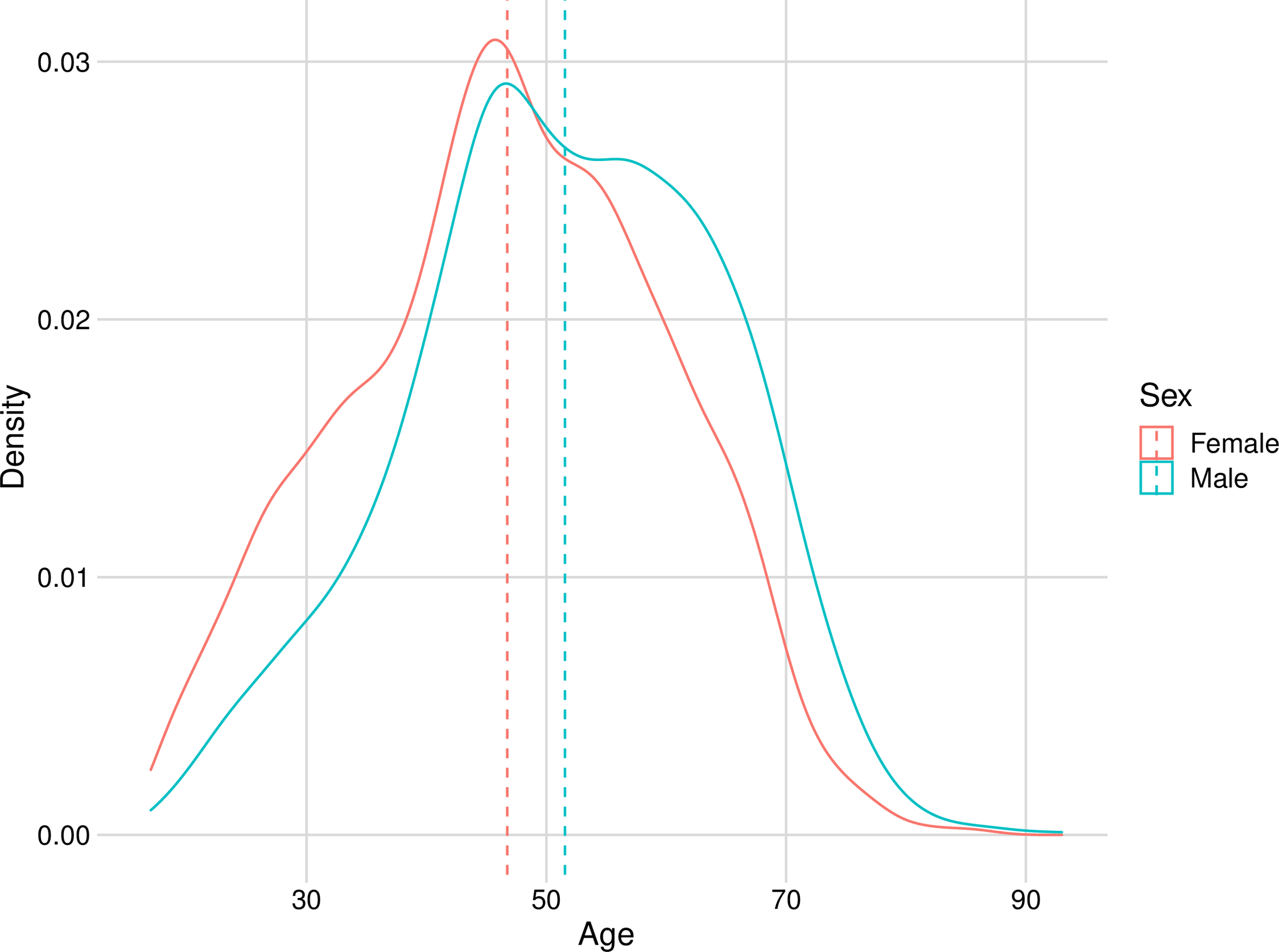

Supplement: S2 Fig — (TIF) [file pdig.0000204.s002.tif]

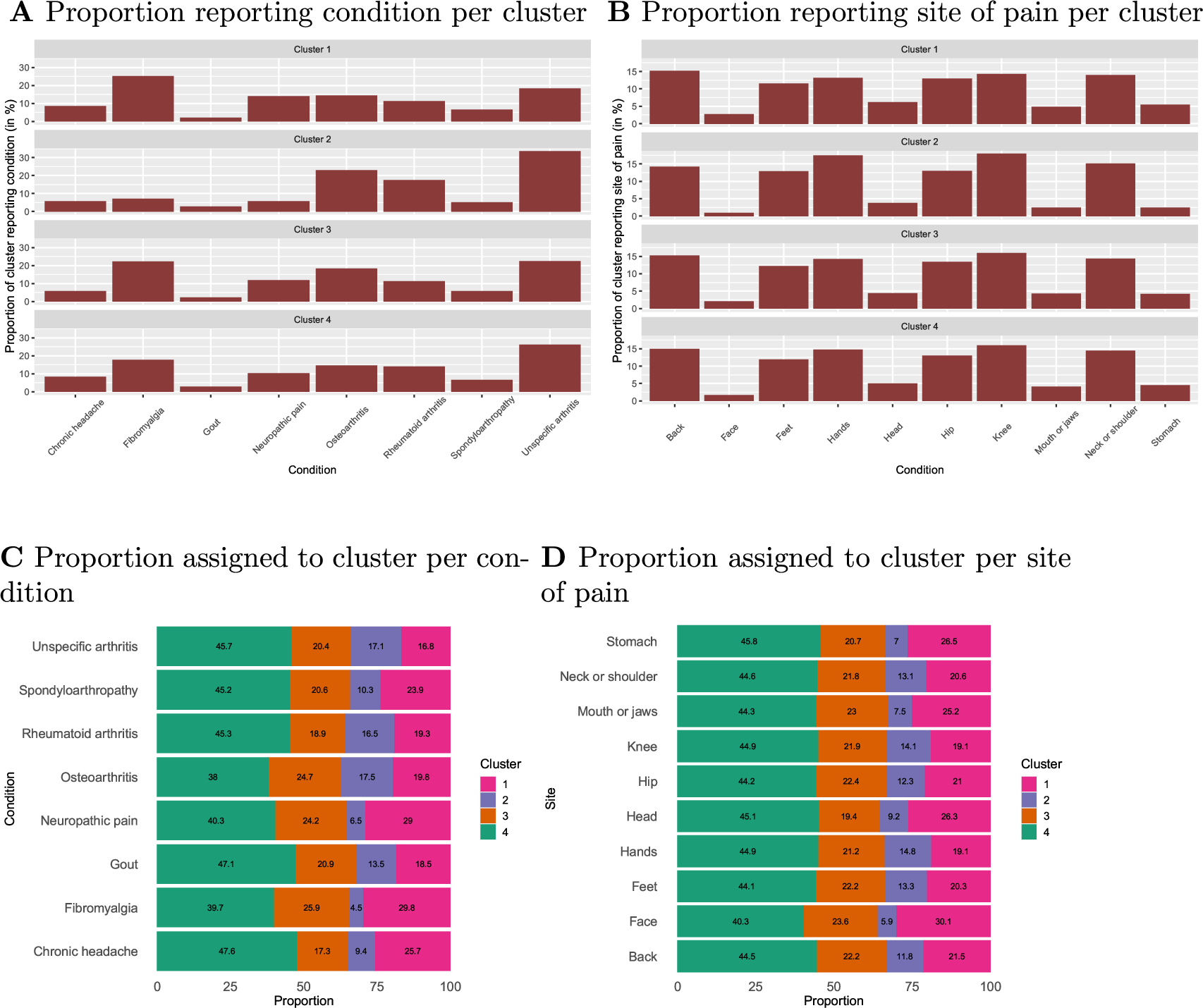

Supplement: S3 Fig — A and B indicate the proportion of participants in a cluster reporting, respectively, a given condition and site of pain. C and D show the proportions of participants with, respectively, a given condition or site of pain who fall into each cluster. (TIF) [file pdig.0000204.s003.tif]

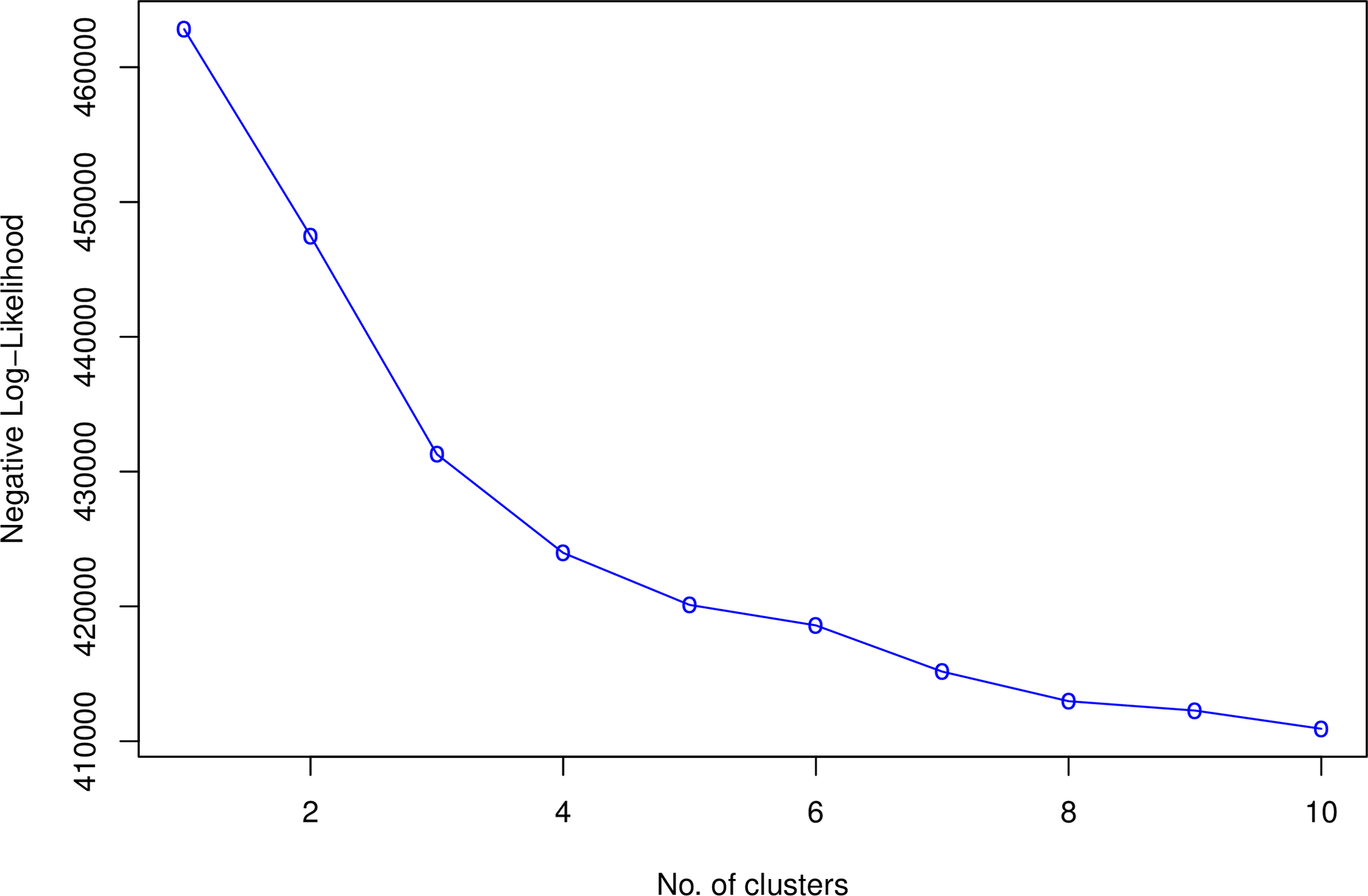

Supplement: S4 Fig — (TIF) [file pdig.0000204.s004.tif]

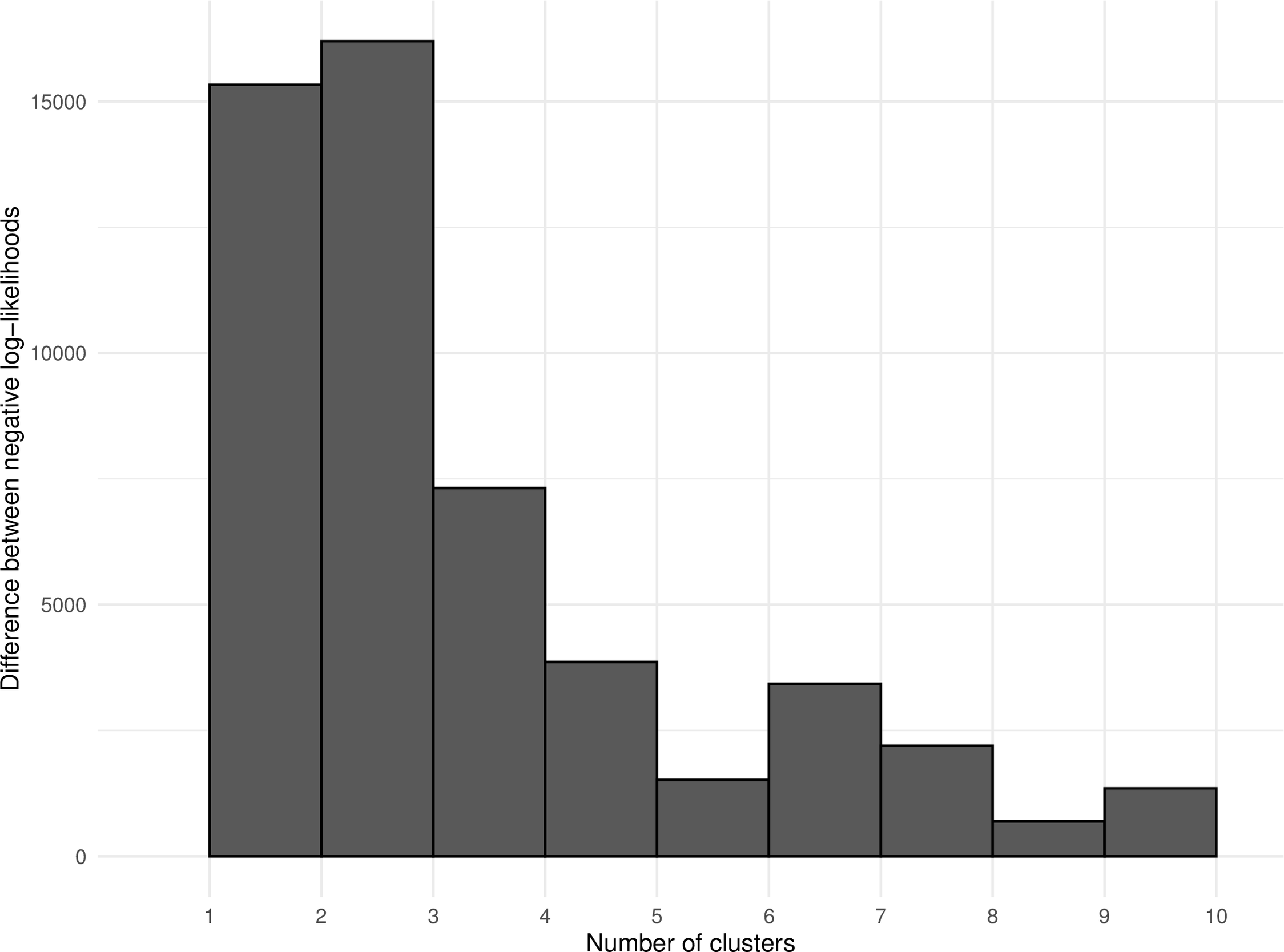

Supplement: S5 Fig — (TIF) [file pdig.0000204.s005.tif]

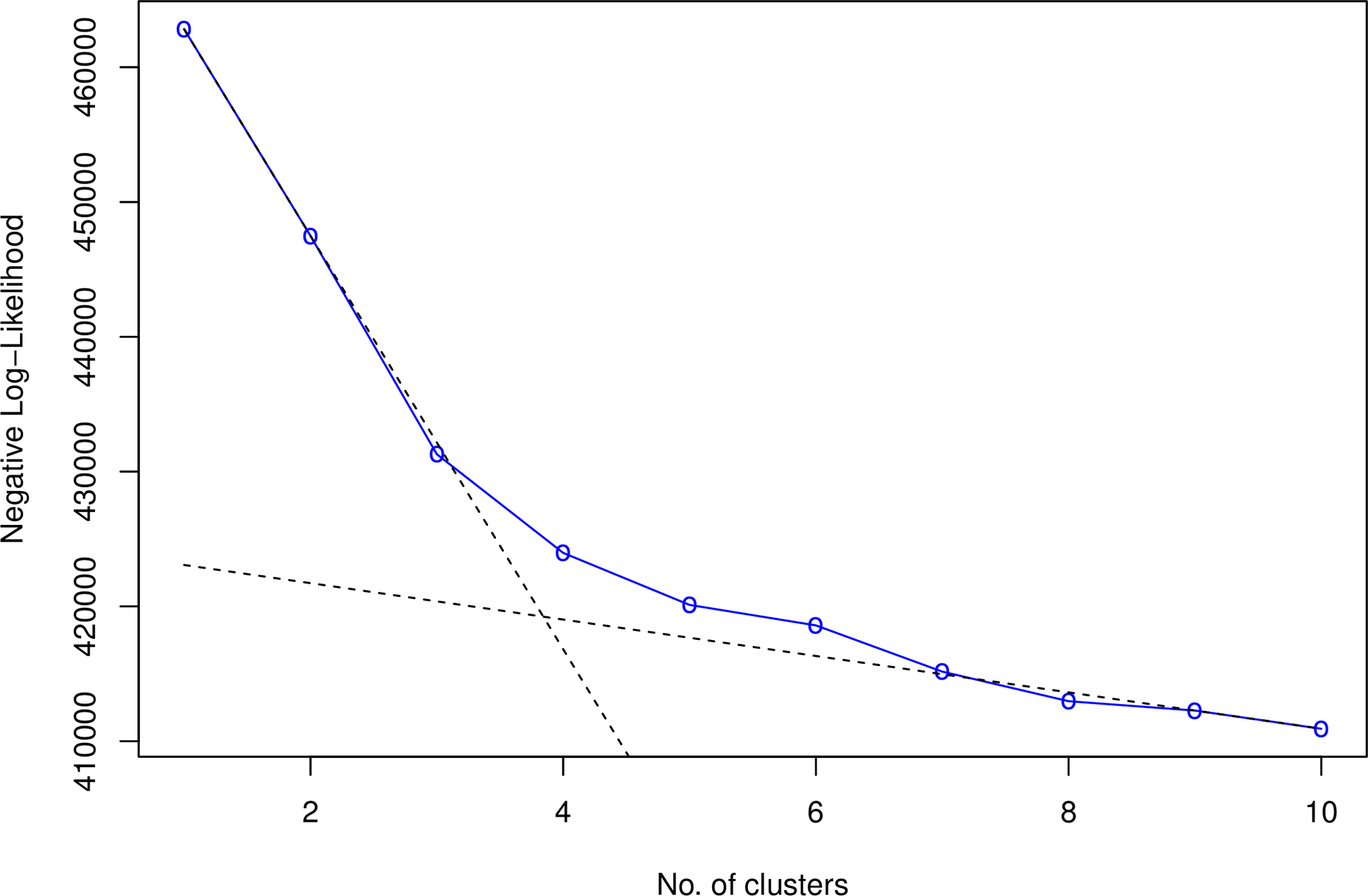

Supplement: S6 Fig — (TIF) [file pdig.0000204.s006.tif]

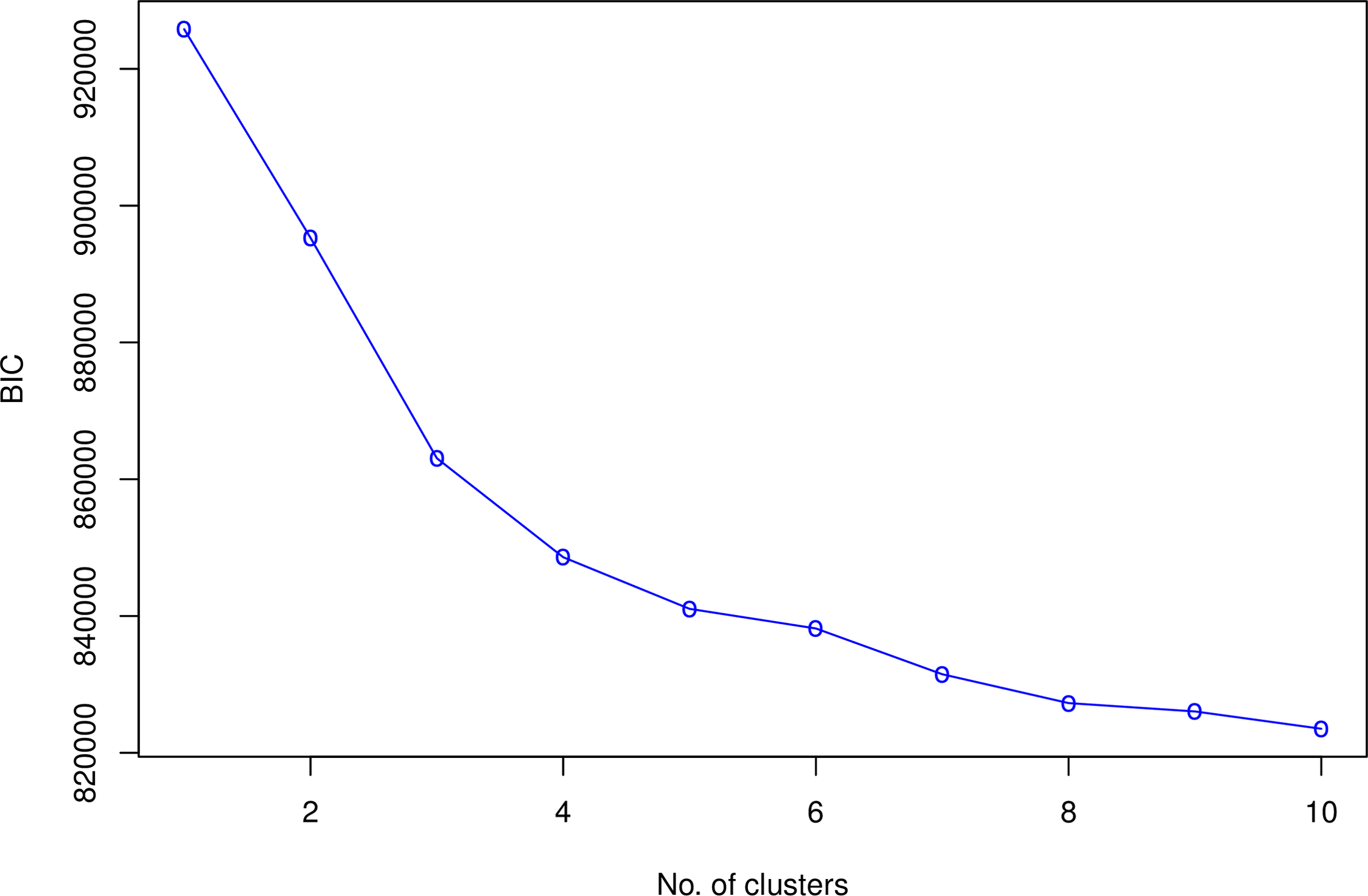

Supplement: S7 Fig — (TIF) [file pdig.0000204.s007.tif]

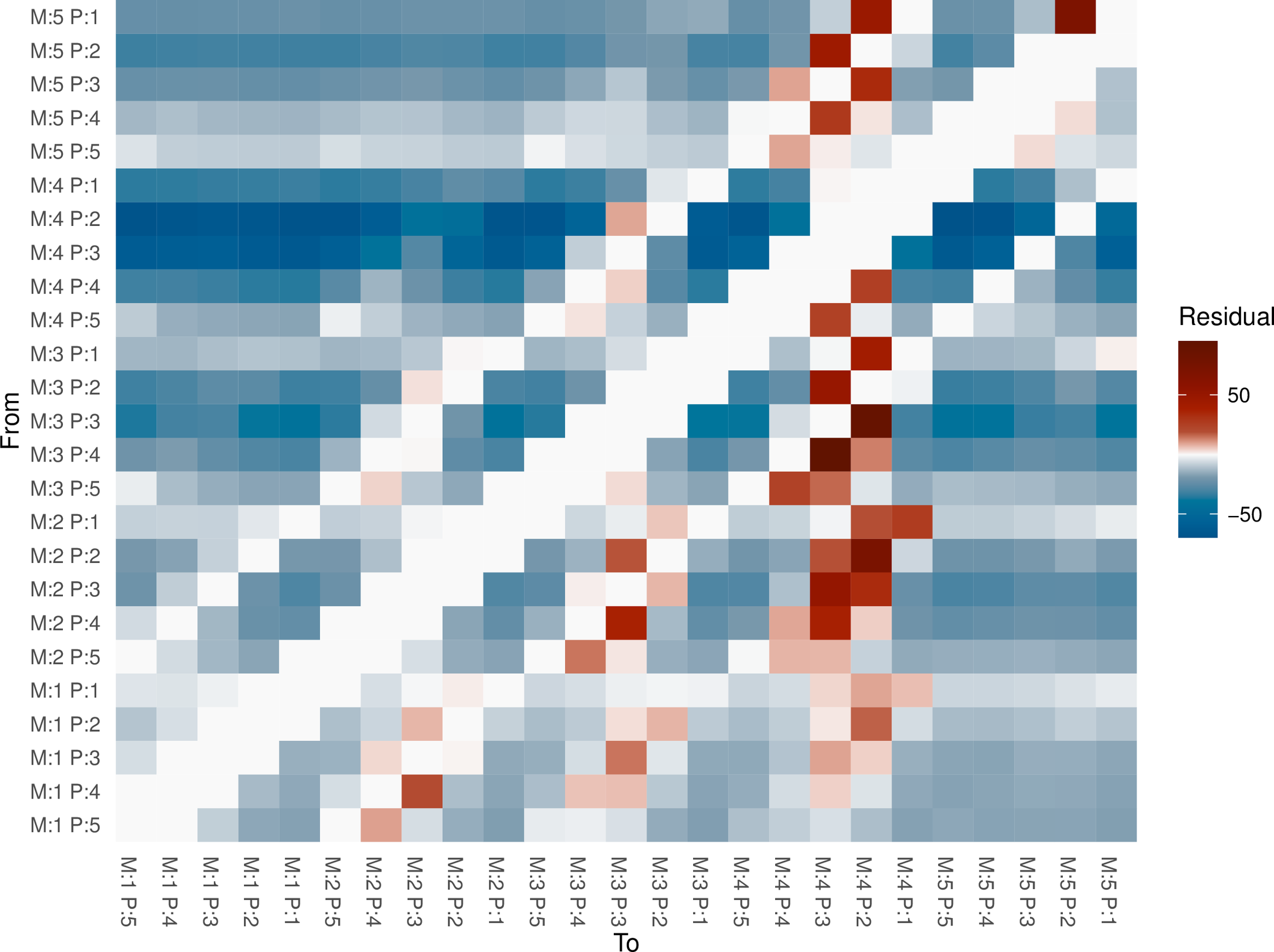

Supplement: S8 Fig — (TIF) [file pdig.0000204.s008.tif]

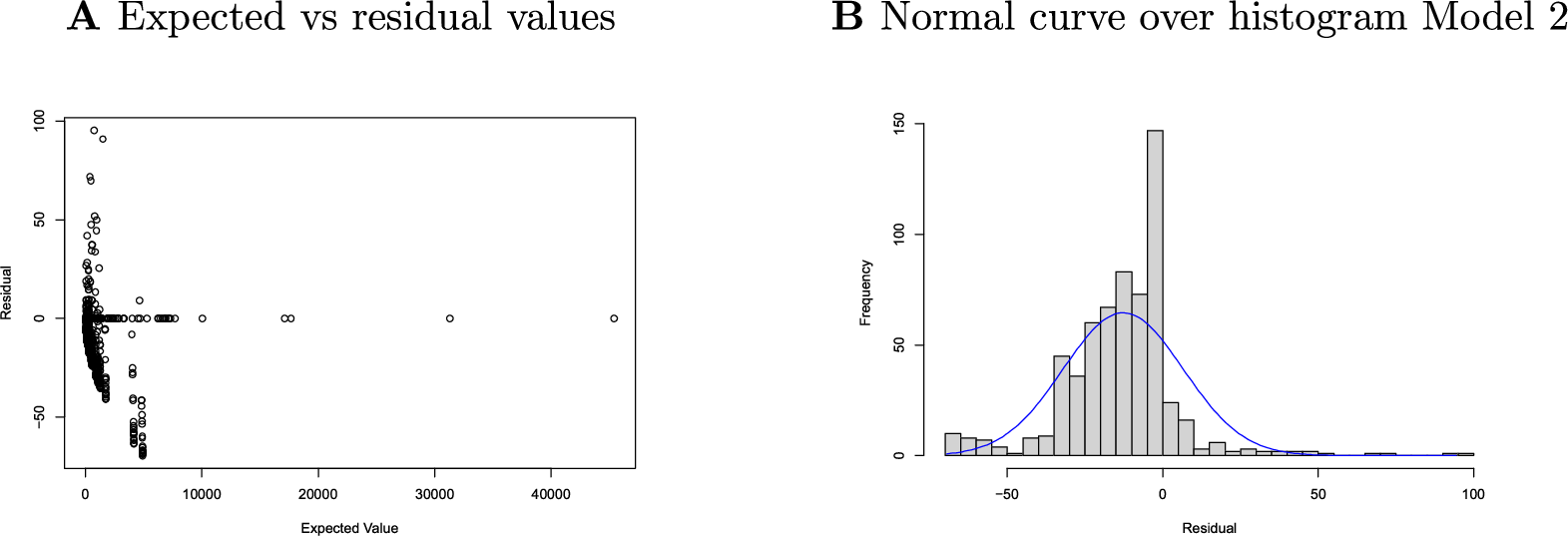

Supplement: S9 Fig — A is the scatter plot of expected values and the residuals. B shows a histogram of the residuals as well as a blue curve giving the probability density function of a normal distribution having the same mean and variance as the residuals. (TIF) [file pdig.0000204.s009.tif]

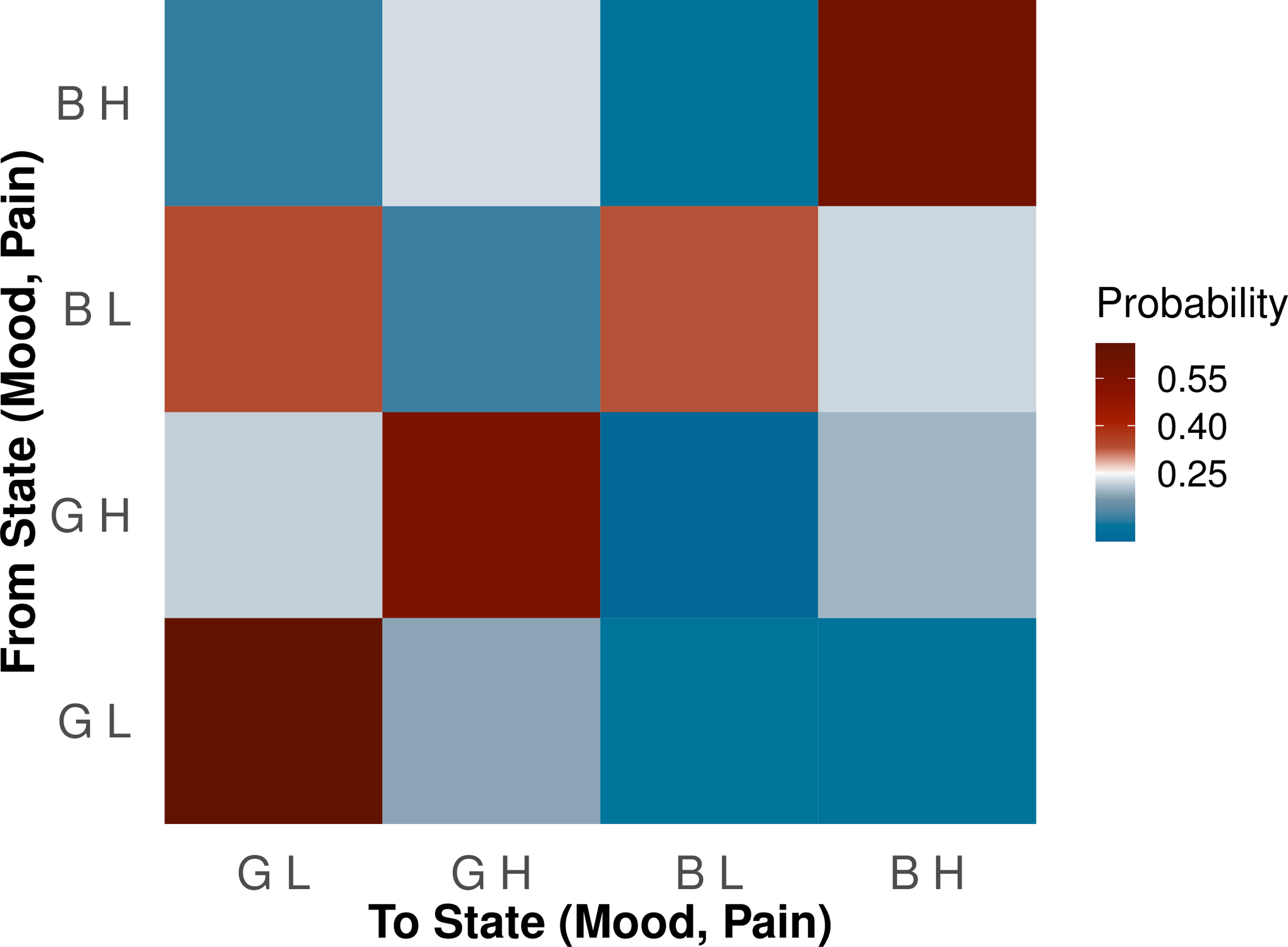

Supplement: S10 Fig — (TIF) [file pdig.0000204.s010.tif]

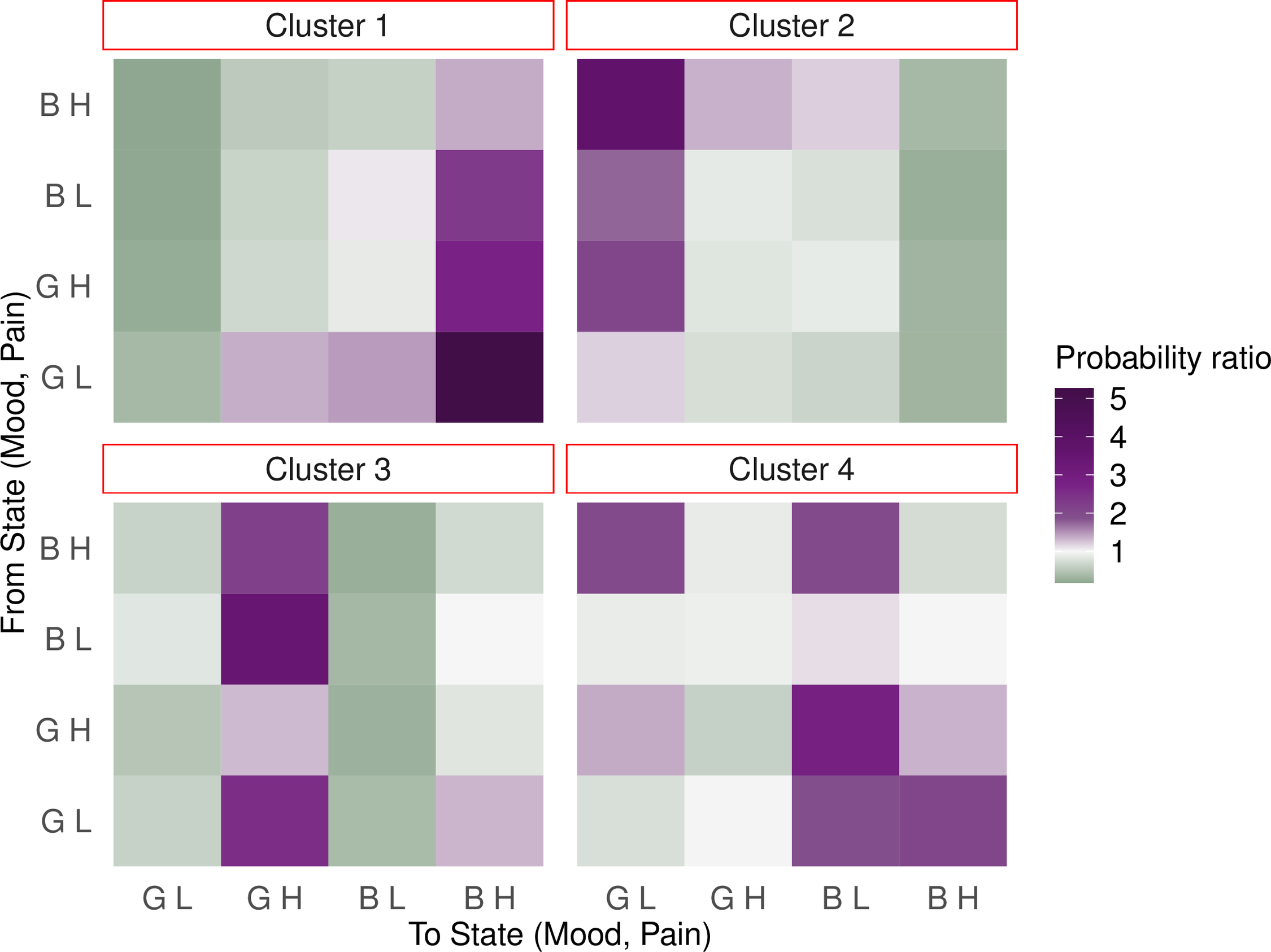

Supplement: S11 Fig — (TIF) [file pdig.0000204.s011.tif]

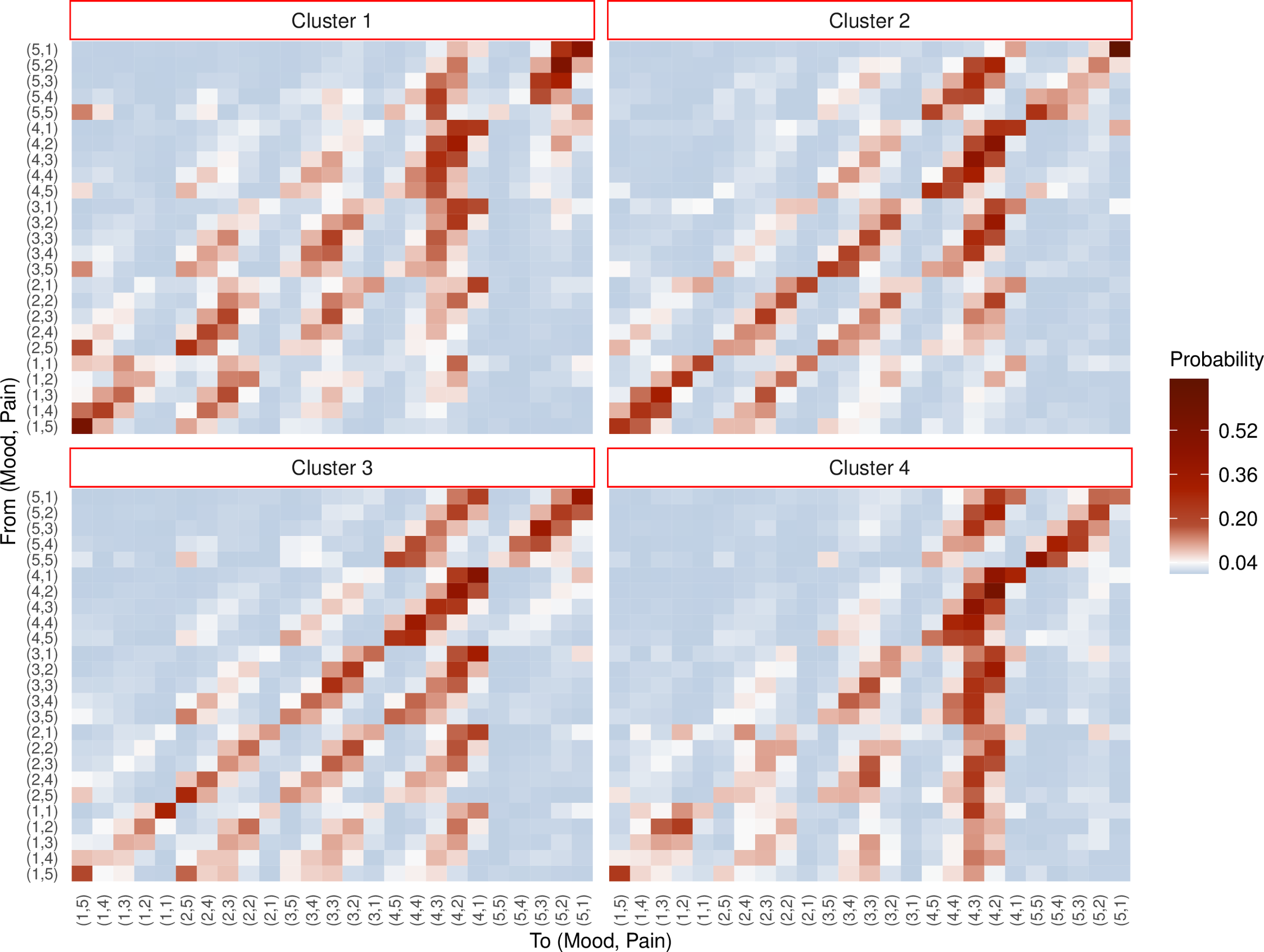

Supplement: S12 Fig — (TIF) [file pdig.0000204.s012.tif]

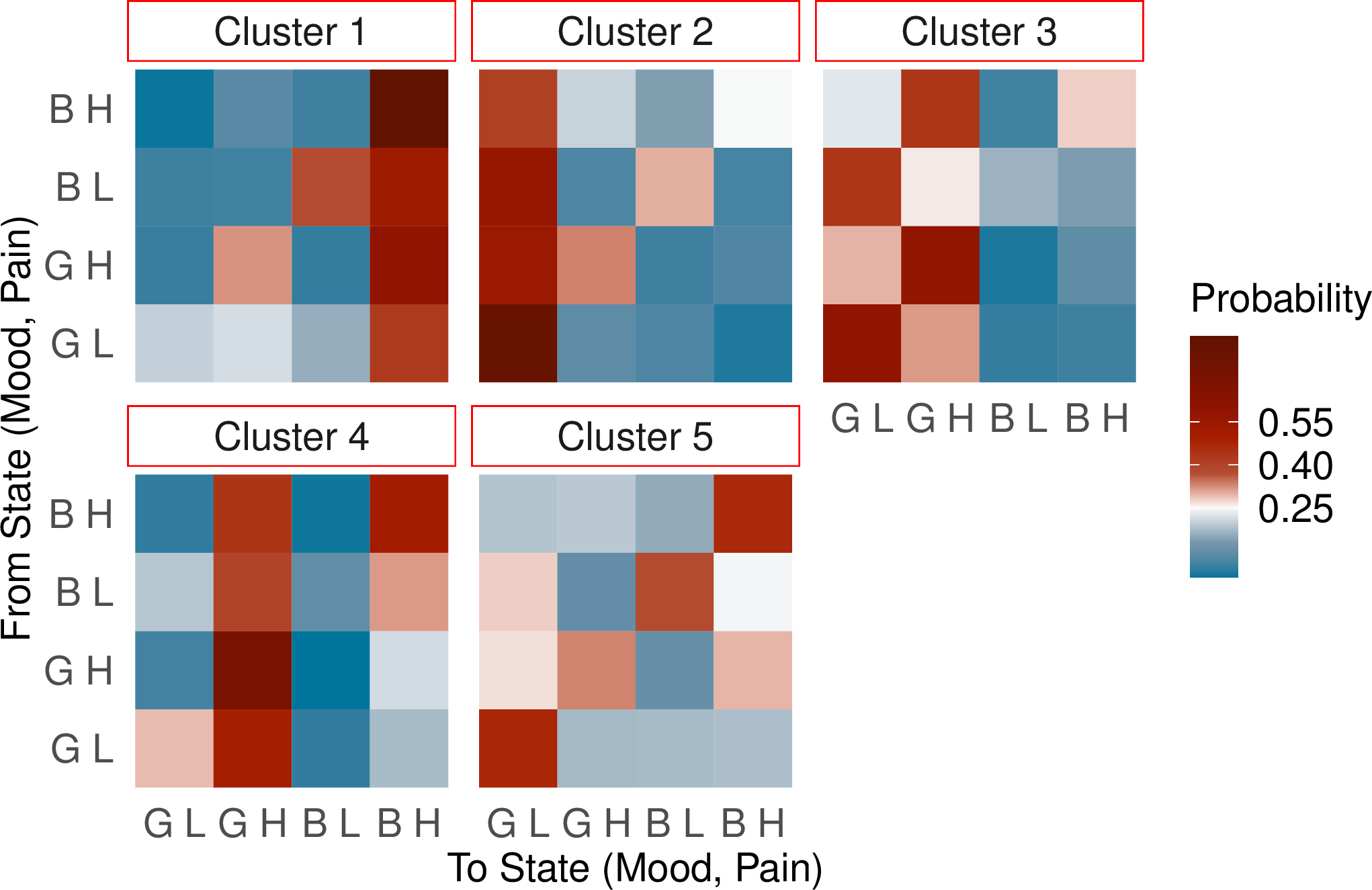

Supplement: S13 Fig — (TIF) [file pdig.0000204.s013.tif]

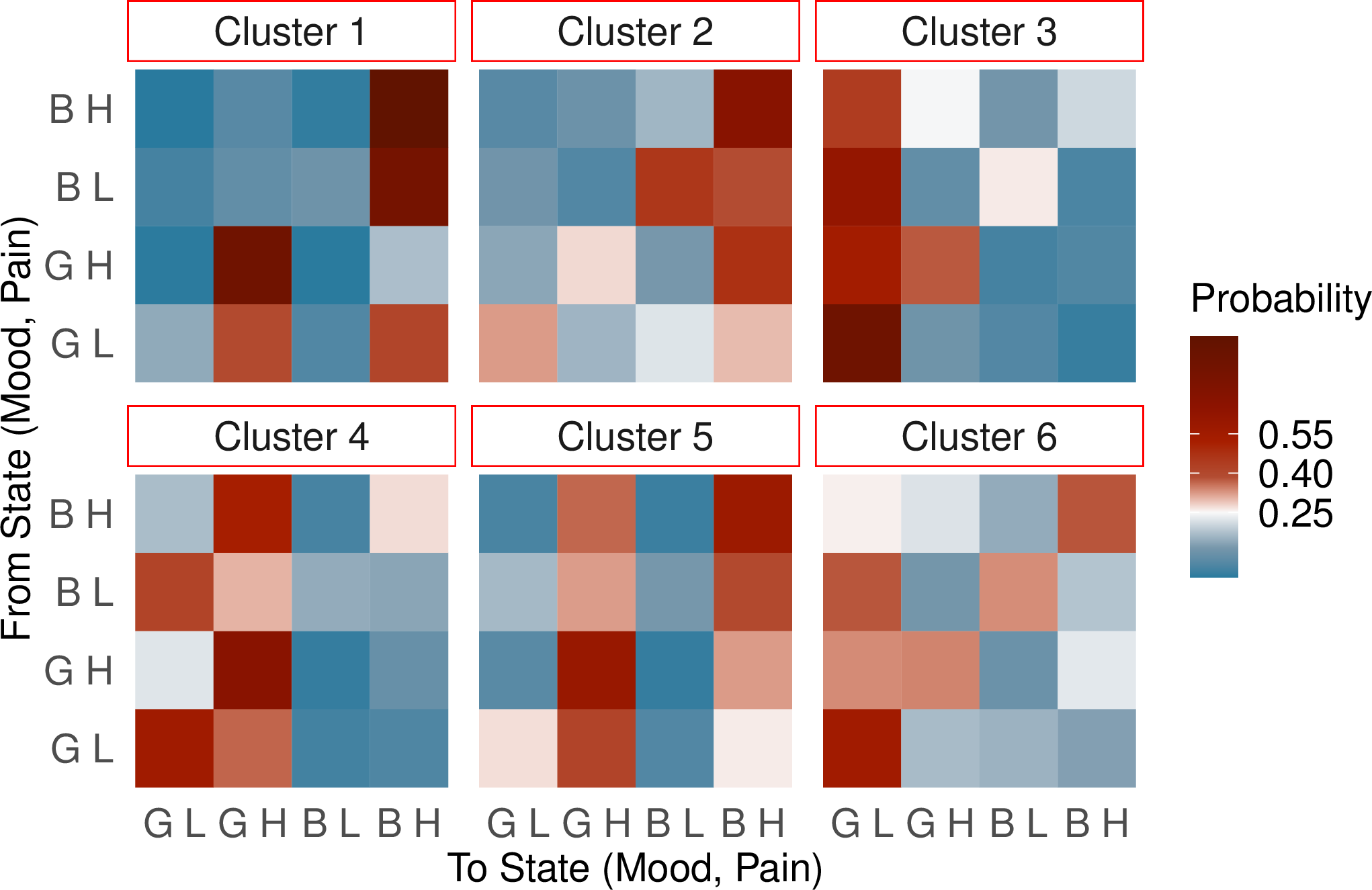

Supplement: S14 Fig — (TIF) [file pdig.0000204.s014.tif]

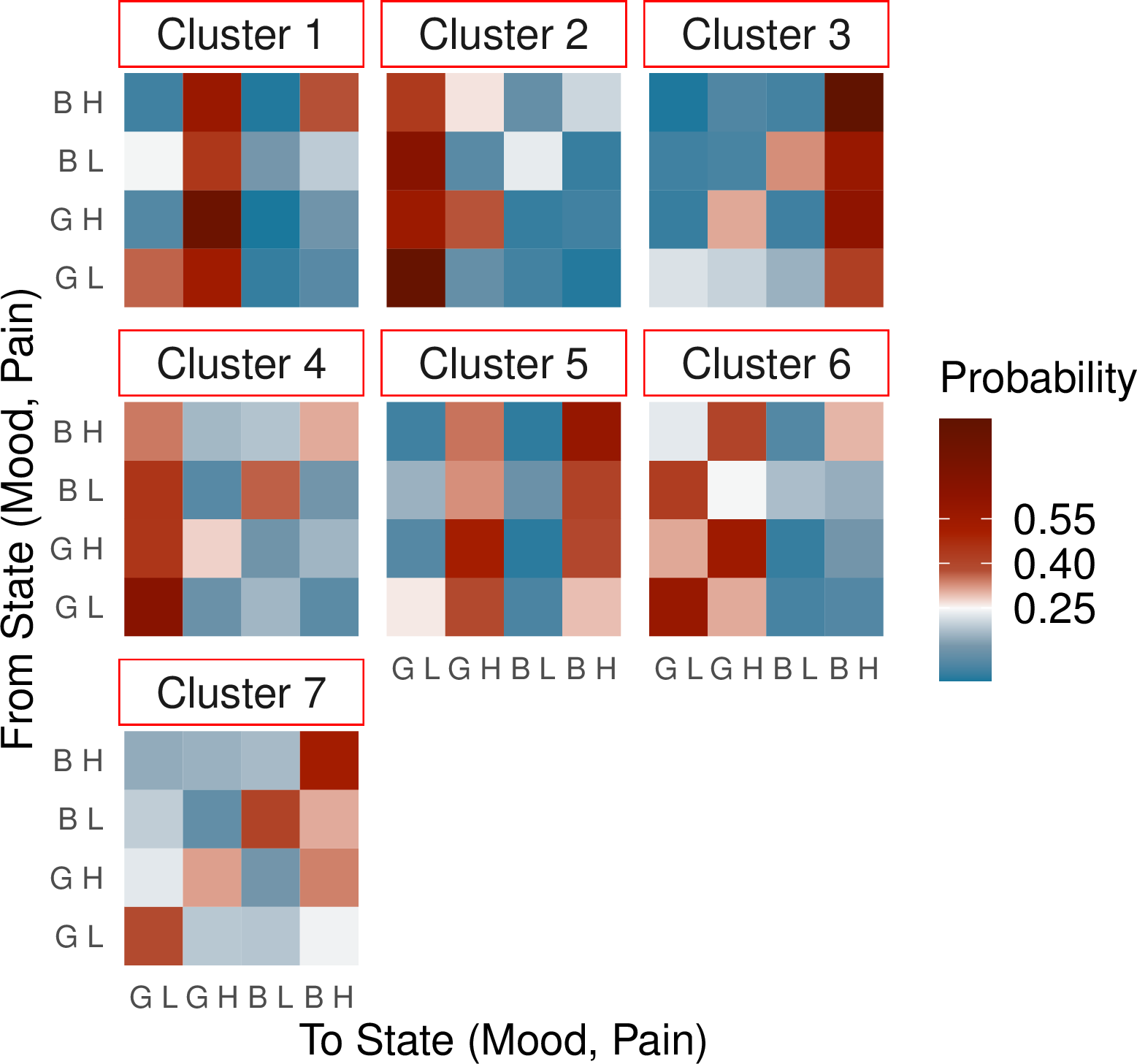

Supplement: S15 Fig — (TIF) [file pdig.0000204.s015.tif]

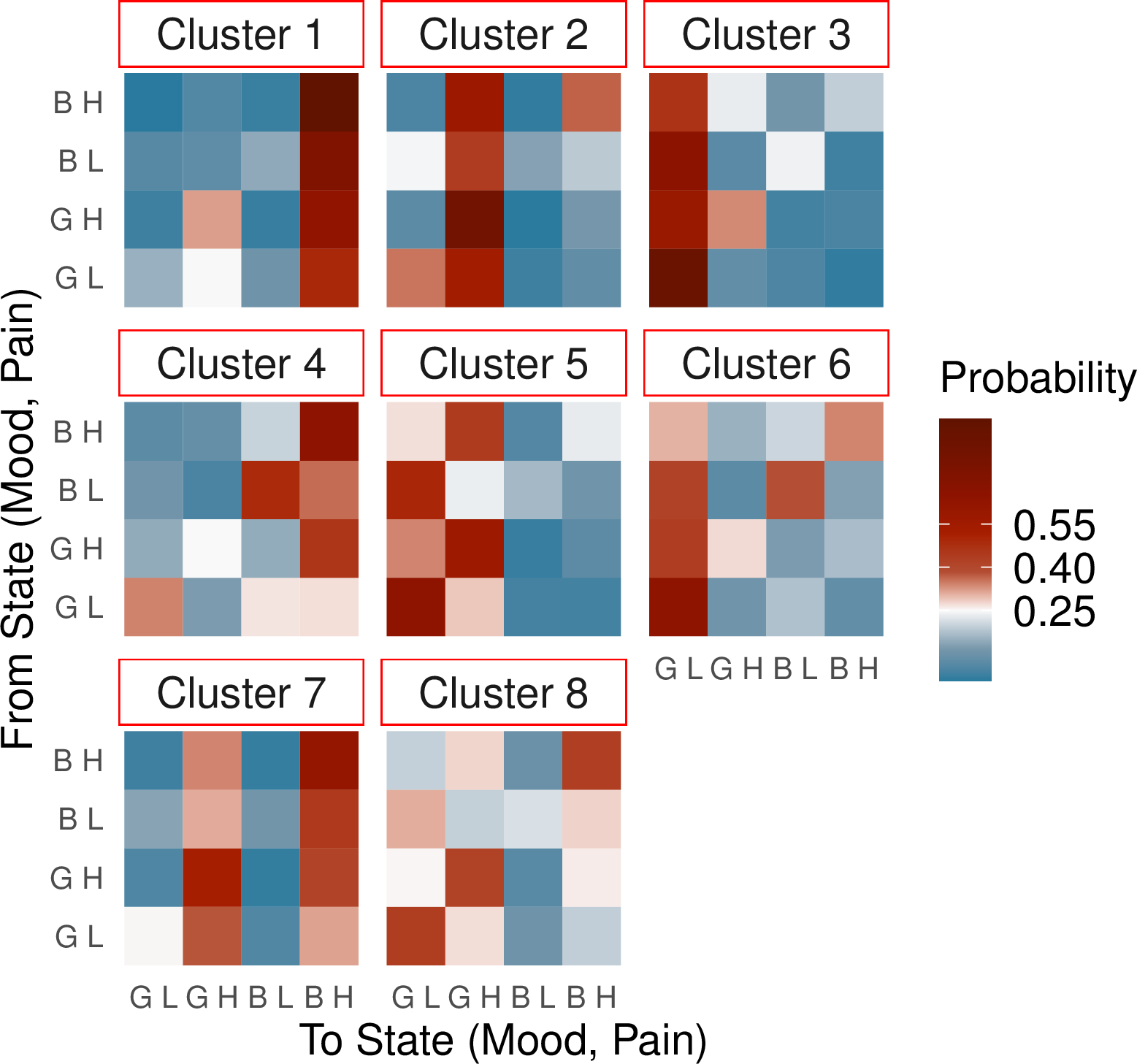

Supplement: S16 Fig — (TIF) [file pdig.0000204.s016.tif]
