# Supplementary material for: Modelling and classifying joint trajectories of self-reported mood and pain in a large cohort study
Source: PLOS Digit Health. 2023 Mar 30;2(3):e0000204. doi: 10.1371/journal.pdig.0000204 (PMC10062665; doi:10.1371/journal.pdig.0000204)
Supplement: S1 Text — (PDF) [file pdig.0000204.s022.pdf]

S 1 Supplementary text

S 1.1 Summary statistics of data and results

S 1.1.1 Main data

Here we provide an overview of the data. Both mood and pain were rated on a five point scale where 1 for mood is the worst score while 1 for pain is the best. Similarly 5 means the mood is the best and pain is at its worst. Mean values of mood and pain are 3.6 and 2.7 respectively. Number of NA's: 344784 in mood and 349760 in pain. After removing these NA's from the data, we are left with 9990 participants instead of 10584. More details about the data can be found at `www.cloudywithachanceofpain.com`.

With five possibilities of each of mood and pain, there are 5 × 5 *i.e.* 25 possible (mood, pain) pairs, which would become the states of our Markov processes, leading to transition matrices with $25 \times 25 = 625$ entries. But instead, we reduce the total number of states by regrouping the scores of mood and pain into good and bad, and low and high categories respectively and then taking pairs of these regrouped scores. For Mood, the Bad (B) scores are {1,2,3} while the Good (G) ones are {4,5}. For Pain, Low (L) is {1,2} while High (H) is {3,4,5}.

For Mood, number of B's and G's are, respectively, 153922 and 288145. For Pain the number of H's and L's are, respectively, 245344 and 196723. The frequencies with which the four (Mood, Pain) states are observed is shown in Fig S1. The frequencies for BH, BL, GH and GL are 113632, 40290, 131712 and 156433, respectively.

Fig S2 gives the overall age distribution of the cohort. It shows that the mean age for the women and men are approximately 47 years and 52 years respectively.

Table S1 and Table S2 give the characteristics of the 9990 study participants included in our analysis.

S 1.1.2 Clustered data

We have also included the clustered heatmaps without any regrouping of the categorical variables in Fig S12. Note that the probability of moving to a state gets reduced from 0.25 (in case of grouped data) to 0.04, which introduces problems of interpretability and generalisation, but can help in understanding the contribution of a transition probability to the grouped clusters, in combination with Fig S11.

We see that within cluster composition of the conditions and sites of pain are both more or less the same across the clusters as shown in Fig S3**A** and Fig S3**B**. The biggest difference which can be immediately noted is that in the composition of conditions in cluster 2, Fibromyalgia and Neuropathic pain are less while unspecified arthritis is comparatively more. Next, when we look at how much of a cluster constitutes a condition, we find the proportions are in sync with the in general sizes of the clusters. Noticeable difference in Fig S3**C** is that cluster 2 amounts to very little proportion of Fibromyalgia, while for site of pain, we find in Fig S3**D**, cluster 2 constitutes very little of site of pain as the face, while cluster 1 takes up a high proportion compared to the rest of its contributing proportions.

Table S3 gives the mean age in years and the response rate in percentage per cluster. Mean age was calculated by taking the average of age in a group, after removing all the NA values. Response rate is the percentage of participants in a cluster who gave their date of birth details.

S 1.2 Definition of the log odds ratio

Table S4 and Table S5 give the log odds ratio of a condition and site of pain respectively in a cluster compared to the other clusters.

In this case, we are considering the log odds ratio as defined for 2×2 contingency tables [41], which is a similar concept to that in logistic regression but not fully isomorphic. To calculate such a log odds ratio, we consider the following contingency table first:

	Cluster	Remaining clusters
Condition	n_{11}	n_{12}
Remaining conditions	n_{21}	n_{22}

Explicitly:

n_{11} is the number of participants with a specific condition in a cluster.
n_{12} is the number of participants with the specific condition not in the cluster.
n_{21} is the number of participants without the specific condition in the cluster.
n_{22} is the number of participants without the specific condition not in the cluster.

The log odds ratio is then given by:

$$L = \log\left(\frac{n_{11}n_{22}}{n_{12}n_{21}}\right) = \log(n_{11}) + \log(n_{22}) - \log(n_{12}) - \log(n_{21})$$

The standard error of this quantity is asymptotically equal to

$$\sigma = \sqrt{n_{11}{}^{-1} + n_{12}{}^{-1} + n_{21}{}^{-1} + n_{22}{}^{-1})},$$

as shown in [41]. Therefore, the 95% Confidence Interval is approximately $L \pm 1.96\,\sigma$.

Similarly we calculate for site of pain by building the following contingency table:

	Cluster	Remaining clusters
Site of pain	n_{11}	n_{12}
Remaining sites of pain	n_{21}	n_{22}

Explicitly:

n_{11} is the number of participants with a specific site of pain in a cluster.
n_{12} is the number of participants with the specific site of pain not in the cluster.
n_{21} is the number of participants without the specific site of pain in the cluster.
n_{22} is the number of participants without the specific site of pain not in the cluster.

And we can then calculate a log odds ratio as for conditions. In general, a positive log odds ratio indicates that the site or condition is more commonly in a cluster, and a negative that it is less commonly so.

S 1.3 Description of the EM algorithm

The matrix $\mathbf{\Gamma}$ is initialised randomly with probabilities chosen such that every row sums to 1. The mixture of Markov chains is then specified by a weight vector $\boldsymbol{\omega}$ of length K in which

$$\hat{\omega}_k = \frac{\sum_{s=1}^{S} \Gamma_{sk}}{S} \tag{1}$$

Using the mixture weights and count matrices, we then estimate the parameters of the per-cluster Markov chains which are transition probability matrices $\mathbf{M}$. For cluster

k, the estimate is

$$\hat{M}_{kij} = \frac{\sum_{s=1}^{S} \Gamma_{sk}\, C_{sij}}{\sum_{k=1}^{K}\sum_{s=1}^{S} \Gamma_{sk} C_{sij}}. \tag{2}$$

The rows of the a participant's count matrix are taken to follow a Multinomial distribution and so define an $S \times K$ matrix of expected likelihoods whose entry Λ_{sk} gives the likelihood of observing participant s's trajectory given that the participant s belongs to cluster k. It is given by

$$\Lambda_{sk} = \prod_{i,j=1}^{n} M_{kij}^{C_{sij}}$$

where we have suppressed a multinomial coefficient that does not depend on the parameters of the mixture model and so does not affect maximum-likelihood estimates. The log-likelihood for participant s and cluster k is thus given by,

$$\log \Lambda_{sk} = \sum_{i,j=1}^{n} C_{sij} \log\left(M_{kij}\right). \tag{3}$$

Using Eq (3), we can specify the algorithm steps as follows:

- **Expectation step:** The expected values of the matrix of class membership probabilities are computed using

$$\widehat{\Gamma}_{sk} = \frac{\omega_k\, \Lambda_{sk}}{\sum_{c=1}^{K} \omega_c\, \Lambda_{sc}}$$

- **Maximisation step:** This involves re-estimating the parameters of the mixture using Eq (1) and Eq (2).

One performs the steps in alternation until the matrix $\mathbf{\Gamma}$ converges. That is, one keeps track of the two most recent estimates of $\mathbf{\Gamma}$ — call them $\widehat{\mathbf{\Gamma}}$ and $\widehat{\mathbf{\Gamma}}'$ — and continues iterating until a convergence criterion such as

$$|\widehat{\mathbf{\Gamma}} - \widehat{\mathbf{\Gamma}}'| < \epsilon,$$

for some sufficiently small ϵ is met.

S 1.4 Choosing the number of clusters

To find the total log-likelihood of the observed data, we made use of the log-likelihood per participant per cluster $\log(\Lambda_{sk})$ as found in Eq (3):

$$\sum_{s=1}^{S} \log\left(\sum_{k=1}^{K} \omega_k \Lambda_{sk}\right), \tag{4}$$

where s denotes the participant and k ranges over the clusters.

Fig S4 shows the negative log-likelihood as a function of the number of clusters. As expected, we see the largest drop between $K = 1$ and $K = 2$, with diminishing returns as K increases. Ideally a quantitative trade-off between model complexity and reducing loss would be made, however most such formal measures do not perform well for large datasets such as the one we consider here, and so here we use the elbow method and assess optimal value of K. To this end, we continue to see relatively big decreases as we increase the number of components to $K = 3$ and $K = 4$. The curve continues falling

after $K = 4$, but as the decreases in negative log-likelihood are modest, following this, 4 clusters is indicated by the elbow method. To make this reasoning clearer, we extrapolated consecutive negative log-likelihood differences in Fig S5 to show that $K = 4$ indeed sits at the crossover between the small- and large-K regimes.

To demonstrate the difficulties in using a quantitative trade-off for data of our scale, we consider the Bayesian Information Criterion (BIC), which is a method to compare statistical models by calculating the information loss between the true and evaluated model by penalising the sample size to address the problem of overestimating the number of parameters [1].

First we compute the likelihood L for the model in consideration. Then, we write the BIC as

$$\mathrm{BIC} = -2\log L + k\log(n)\,,$$

where $\log L$ same as that given in 4. k is the total number of parameters and n is the number of observations.

For the problem in question, $k = K \times \mathrm{size}(\mathbf{T}) \times (\mathrm{size}(\mathbf{T}) - 1) + K - 1$ where K is the total number of clusters and $\mathbf{T}$ is the transition probability matrix therefore, for the 4-state Markov mixture models, we get $k = K \times 4 \times (4 - 1) + K - 1 = 13K - 1$. Number of observations is the total number of transitions which is 432077. We select the optimal number of clusters in the similar way as that done for negative log-likelihood above. Its plot is given in Fig S7. However, we do not see much difference from the previous model selection plot in Fig S4. This is because in comparison to the large size of the dataset, the penalty in BIC is too small and does not give a criterion value much different from the negative log-likelihood. It is common to have BIC and other criteria to keep on decreasing in case of large datasets and other authors such as [2] used similar reasoning to ours when performing model selection.

Additionally, we performed clustering into 5 to 8 components as shown in Fig S13, Fig S14, Fig S15 and Fig S16. We see that for $K > 4$, some clusters are very small, involve repeated patterns, and are hard to interpret (in contrast to the discussion as in the Discussion section of the main paper) also leading us to prefer $K = 4$.

S 1.5 Residual analysis: A second model

In order to fit a better model, let's us go back to the observed transition matrix plotted in Fig 1**A**. The observation that the diagonal elements are high has already been incorporated into the model described by Eq (1). If we look at the heatmap more carefully, we can see that there are more dark bands indicating high probabilities in certain off-diagonal regions as well.

Let m and p denote the original mood and pain scores respectively. The states are in the pairs of form (m, p) where, $m, p \in \{1, 2, 3, 4, 5\}$. Since people tend not only to remain in the same state, but also to move a single step up or down in either mood or pain, it is interesting to extend the simple model of Eq (1) to capture these features. Let the probability that a person remains in the same state (m, p) be $\pi_{m,p}$ and the probabilities that they move to a state with $p \pm 1$ be $\pi_{m,p\pm1}$ and probability of moving to a state with $m \pm 1$ be $\pi_{m\pm1,p}$.

Assuming independence holds, the model is re-defined using the following distribution. $P_{(m,p),(m',p')}$ is the probability of people moving from state (m, p) on a day to (m', p') the next day. For $m, p \in \{1, 2, 3, 4, 5\}$,

$$P_{(m,p),(m',p')} = \begin{cases} \pi_{m,p} & \text{if } (m',p') = (m,p) \\ \pi_{m,p\pm1} & \text{if } (m' = m) \text{ and } (p' = p \pm 1) \\ \pi_{m\pm1,p} & \text{if } (m' = m \pm 1) \text{ and } (p' = p) \\ \text{uniform} & \text{otherwise} \end{cases} \tag{5}$$

The new model is thus that the probabilities for staying at the same state or moving to states whose mode or pain scores differ by 1 agree with those implicit, but transitions to all other states are equally likely. When we overlay a standard normal curve on the histogram of standardised residuals, as shown in Fig S9, we find once again that the residuals do not appear to be normally distributed.

Using the same standardised residual formula as in Eq (2), the heatmap of the residuals shown in Fig S9 is obtained. Comparing it with Fig 1, the first noticeable difference is that the range of residuals for the new model has decreased, indicating a better fit. Also, the diagonal region connecting the top left to bottom right has smoothed out a bit.

We could in principle carry on, constructing models of increasing complexity and reducing the largest residuals until those that remain have the expected, near-normal distribution. But this modelling effort was only meant to be exploratory: our main goal was the clustering analysis as discussed before.

Fig S9**A** compares the expected values and residuals obtained from Eq (5), and Fig S9**B** shows how the normal distribution curve fits the histogram of residuals.

S 1.6 Clusters

Transition probability matrix based on the regrouped states is given in Fig S10.

Before clustering, we take a look at the transition probability matrix again, but with the new states where we have regrouped the states into two categories, Good (G) and Bad (B) for mood, and Low (L) and High (H) for pain. We see trends similar to those in Fig 1**A**, where the probability to remain in any given state is high. Additionally, here we can also see that probability of moving from (Mood, Pain) state (B, L) to (G, L) is high.

Once clustering is done, in Fig S11 we note the distribution of transitions amongst the clusters. Here, the sum of probabilities for a particular transition across clusters add up to 1.

S 1.7 Computing the shift for interventions

Here, we show how we have shifted the transition matrices to intervene with either improving mood or improving pain. This is a bespoke method to find a shift in the given scenario, designed to ensure that we follow the laws of probability. This need not be the optimal solution and in general it would be preferable to build a model to incorporate intervention effects by parameterisation of the state transitions as sets of odds ratios that could be calibrated to studies. To our knowledge, building such a general model for Markov chains is an unsolved problem in discrete multivariate statistics and so we outline the problem-specific approach we adopt in this work below.

Step 1: We first calculate an intermediate result α by taking the maximum of the maximum of the probabilities of transitioning from bad mood to good mood over the clusters:

$$\alpha = \max\{\Pr(\text{mood tomorrow} = G \mid \text{mood today} = B \text{ and cluster} = k)\}\,, 1 \leq k \leq K\,.$$

Step 2: Given α, we calculate $\beta = (1 - \alpha)/2$. Then our new probabilities become

$$\text{Pr}'(\text{mood} = \text{good tomorrow} \mid \text{mood} = \text{bad today}) = \text{Pr}(\text{mood} = \text{good tomorrow} \mid \text{mood} = \text{bad today}) + \beta.$$

To ensure that the probabilities add up to 1, we impose $\text{Pr}'(\text{mood} = \text{bad} \ \& \ \text{pain} = \text{low} \mid \text{mood} = \text{bad}) = 0.8 \times \text{Pr}'(\text{mood} = \text{good} \mid \text{mood} = \text{bad})$ and $\text{Pr}'(\text{mood} = \text{bad} \ \& \ \text{pain} = \text{high} \mid \text{mood} = \text{bad}) = 0.2 \times \text{Pr}'(\text{mood} = \text{good} \mid \text{mood} = \text{bad})$. We split in the ratio of 4:1 to give lesser importance to the least-ideal state BH. For our data, we get the approximate maximum value of β as 0.15. So we shift by 0.15 and compare with the clusters. In a similar way, we calculated β_P.

References

1. Dorea CC, Goncalves CR, Resende P. Simulation results for Markov model seletion: AIC, BIC and EDC. In: Proceedings of World Congress on Engineering and Computer Science. vol. 2; 2014. p. 899–901.

2. Yin P, He Q, Liu X, Lee WC. It takes two to tango: Exploring social tie development with both online and offline interactions. Statistical Analysis and Data Mining: The ASA Data Science Journal. 2016;9(3):174–187.
