# Supplementary material for: Modelling and classifying joint trajectories of self-reported mood and pain in a large cohort study
Source: PLOS Digit Health. 2023 Mar 30;2(3):e0000204. doi: 10.1371/journal.pdig.0000204 (PMC10062665; doi:10.1371/journal.pdig.0000204)
Supplement: Attachment — Submitted filename: ResponseToReviewers.pdf [file pdig.0000204.s023.pdf]

MODELLING AND CLASSIFYING JOINT TRAJECTORIES OF SELF-REPORTED MOOD AND PAIN IN A LARGE COHORT STUDY: RESPONSE TO REVIEWS

We would like to thank the editor and reviewers for their constructive comments on our first submission that have allowed us to improve our manuscript, and now detail how we have responded to these below as well as in an annotated version of the revised manuscript. Answers are written in bold.

EDITOR

1) Please provide the clarifications the reviewers request, mostly in the supplementary material.

Please see detailed responses to reviews below.

2) Please make your code available in a public repository, and encourage the data-controller to make suitable synthetic data available for easier knowledge-transfer of your methods/code.

The code is available at https://github.com/rajenkidas/EM-clustering-on-Markov-Chains; the data controllers have had some technical problems with arranging this but expect the data to be available via trusted environment quite soon and these facts are now stated in Section 2.

3) Align your clarification of model selection with the code you publish, linked to from the supplementary material.

Please see detailed responses to reviews below.

4) Clarify the cohort definition/characterisation with more information about https://www.cloudywithachanceofpain.com in the supplement.

We now state this in Section 2 and supplementary tables S2 and S3 as detailed below.

5) Optionally, you may wish to elaborate on the use of (variable) longitudinal observation designs to distinguish patterns of resilience to mood/pain worsening from patterns of susceptibility, with potentially earlier tipping points and different sequencing.

We agree with this as an important direction for future research, however we believe that addressing it requires substantially different methodology from that used here and so we elaborate on this in the Discussion of the manuscript.

REVIEWER 1

1) the authors don't provide detail on whether or not the participants were self-selecting and self-reporting diagnoses. This would help situate the paper for clinically-focused readers who might ask whether or not the participants were a clinical population (i.e. any diagnoses of mood disorders and/or confirmed diagnoses of pain syndromes / musculo-skeletal / rheumatoid disorders). The introduction helpfully describes the pain <-> mood bidirectional association, so it would be worth highlighting if this is a "clinical population" or a group of people

self-selecting to trial monitoring their mood <-> pain symptoms. Alternatively, in the Limitations sections, the authors could clarify if this can be considered a clinical population or not. and what is known about participants' diagnoses.

Participants were self-selecting and self-reporting diagnoses, and so we agree that it is important in interpretation of results to note that they are not a clinical population. We now provide further details in Section 2, and have added supplementary Tables S2 and S3 giving details about the demographics on the participants' diagnoses and sites of pain.

2) I struggled to understand how the "intervention" (which appears to simulate changes in individual cluster's transition probability matrices) simulations were implemented -- for example, after fitting the k = 4 model, from my reading, it seems the authors then set the beta_P and beta_M values (in addition to the transition probability matrix contraints -- constants 0.8 and 0.2 -- to impose an asymmetric transition probability BH and BL from BG and BL) so that beta_M < 0.15 and beta_P < 0.2. If the intention is to show that the model could allow one to simulate intervention, then I would either expand on this in the main text, or if this is less important, I would move to supplementary information -- as it stands, I couldn't find an a priori reason why this simulation was included in the main manuscript (especially given they chose the constants/penalties and beta values themselves). I think I understand the what the authors are attempting to show, but my overall impression is that this is somewhat arbitrary and I'm not entirely clear that it adds much to the results presented.

Details on selection of Beta (shift for transition matrix) have been provided in the supplementary Section S.1.7. We tried to come up with a mathematical formulation of such interventions that would avoid arbitrariness for the reasons the reviewer says, in particular through parameterisation of the state transitions as sets of odds ratios that could be calibrated to studies. This turns out to be an open problem in discrete multivariate statistics, and so beyond the scope of the current work. Our numerical experiments did indicate, however, that the results are not terribly sensitive to any reasonable choice of intervention. The positive reason for including any intervention is that we believe it is important in digital health work to show that there is the potential that any effect sizes shown are large enough to impact on treatments rather than rely on generic statistical learning measures of fit, although we would indeed seek to avoid over-interpretation of this section.

3) Model selection: The authors argue for a qualitative "elbow" in the negative log-likelihood implying a solution with clusters k = 4 (Figure S4, section S.1.4) and that "4-component mixture had certain natural interpretations". While this is reasonable, I wondered why the authors did not select clusters using a method that explicitly penalises for model complexity (i.e. the number of parameters) such as BIC ? If the data supports a k = 4 cluster model, one would expect the resulting penalised neg log lik to be parabolic, with a minimum at 4 -- if this is the best compromise between under/over-fitting the number of clusters ? Perhaps the authors could comment on this in Suppl Info.

We agree with the preferability of a formal criterion, however AIC and BIC under-penalise complexity for large datasets, and we provide a plot showing

that indeed our data suffers from this problem, so we have used the elbow method without explicit penalisation but rather as a less formal method to see when 'diminishing returns' of additional complexity start. We now provide lines of early and late gradients of the elbow plot showing that by this criterion 4 clusters is well motivated, as well as heatmaps for extra clusters showing their difficulty in interpretation above K=4.

More details have been added in section S.1.4 of the Supplementary Material.

REVIEWER 2

1) For how many days were participants expected to respond on the App? What happened if a person missed one day but then continued to fill in the app?

The participants were expected to respond to the app for the complete period of the study which was 1 year 3 months, January 2016 to April 2017. Average (rounded off) length of trajectories is 44. These details are provided in Section 2.1.

If they missed one day, the value corresponding to the self-reported variable, mood or pain in this case, gets recorded as NA (not applicable). For this study, we removed all the NA values. So, if somebody skipped a day, that is not currently reflected in the sequence of states considered. This has been stated in Section 2.1 as "However, participants did not always report all the data daily so we considered only those (Mood, Pain) states where both the values are available, leaving us with N = 9990 participants for our analysis."

Dealing with missing values remains a big problem so we have not addressed it in this study, but have specified the existence of missing values in our data in the discussion of the paper.

2) How were the odds ratios in tables S2 and S3 calculated? Something like a multinomial model? Can this be clarified in the methods section please?

The method is described in the supplementary S.1.2. Unfortunately, this is a case where "log odds ratio" is applied to two closely related but subtly different concepts and the method we use comes from the 2x2 table not logistic regression, as is now explicitly stated.

3) The choice has been made to identify 4 clusters. However, in Figure S4, more clusters provide better log-likelihood. The justification is that the reduction is not much once you have more than 4 clusters. But this is a bit arbitrary. It would be good to discuss this a little more and describe what impact this might have. How strongly should we believe that there are four patterns of behaviour for example? In say the 8 or 10 cluster model, do you get lots of similar clusters, or do you find additional patterns to the ones you have identified? The abstract says that you have discovered four distinct patterns – how reliable is this?

The question of cluster number selection was raised in Point 3 by Reviewer 1 – please see response above. In response to the specific questions, our level of certainty in 4 is lower than it would have been if we had a more formal criterion than the elbow method, however we do not see new patterns if clusters are added so are moderately confident in reporting 4 as the appropriate number.

4) What impact do you think reducing the states to 2 per variable has? It's certainly simpler, but do you think it loses useful information on trajectory?

It does lose information and especially in cases where lot of changes within a dichotomised group happen, we fail to observe that.

But, given the approach of assuming it to be a Markov model, taking the number of states as 2 is rather, we feel, is better to get an overall picture. Work is underway to generalise this – however the big problem is that MLEs for larger classifications are badly behaved and so this needs methodological development beyond application of the EM algorithm.

We have, however, added Figure S12 to Supplementary for the ungrouped states to address the issue of state grouping within the scope of the current paper.

5) How reasonable is the Markov assumption in equation 3? I wonder the next state being only dependent on the previous state (and not how it got there) is unlikely to hold – especially in the 25-state model. I suspect this may be less of an issue in the collapsed model presented here with only 2 categories for each of pain and mood. But in the 5 original categories might it not be the case that someone in state 3 was more likely to go to state 4 if they had shown a constant rise from state 1 through to state three than if they had just arrived in state 3 from state 4?

We agree that the system is unlikely to exhibit true memorylessness in the case of consider a 25-state model, and work is underway where the Markov chain assumption of this kind is collapsed. The main benefit of using a Markov chain is that this model can make out of sample predictions, but at the cost of making assumptions that may not hold.

We agree that the Markov assumption is likely to be worse for larger numbers of categories, although while clustering, the transition probabilities at the end points of each of the clusters get affected by the same condition so the clusters already take into account of the restrictions at the extreme points.

We add the Markov assumption now as a limitation in the discussion.

6) The clustering of Markov processes described in this paper is a neat idea. Setting this problem up as a set of states and looking at transitions between them is certainly a natural way to think. I do find clustering of transition matrices a little hard to conceptualise though. There are other ways of doing longitudinal clustering that might work in this setting. For example, latent class trajectory models (Proust-Lima, C., Philipps, V. and Liquet, B., 2015. Estimation of extended mixed models using latent classes and latent processes: the R package lcmm. arXiv preprint arXiv:1503.00890.) or multivariate mixture models (Komárek, A. and Komárková, L., 2013. Clustering for multivariate continuous and discrete longitudinal data. The Annals of Applied Statistics, 7(1), pp.177-200.). I think these would be suitable for your problem (specially with binary pain and mood scores). I'm not proposing redoing any analysis, but I think it would be useful to describe the relative methods of your approach compared to these other examples of approaches to doing essentially the same thing.

Thank you recommending these papers. These have been included in the discussion (Section 4) as potential future work.

In summary, we hope that the Editor and Reviewers are satisfied with our responses to their comments, which we believe have improved the submission.

Best wishes,

Rajenki Das

Corresponding Author

8th January 2023

COMPLETE LETTER

PDIG-D-22-00293
Modelling and classifying joint trajectories of self-reported mood and pain in a large cohort study
PLOS Digital Health

Dear Dr. Das,

Thank you for submitting your manuscript to PLOS Digital Health. After careful consideration, we feel that it has merit but does not fully meet PLOS Digital Health's publication criteria as it currently stands. Therefore, we invite you to submit a revised version of the manuscript that addresses the points raised during the review process.

Please submit your revised manuscript within 30 days Dec 18 2022 11:59PM. If you will need more time than this to complete your revisions, please reply to this message or contact the journal office at digitalhealth@plos.org. When you're ready to submit your revision, log on to https://www.editorialmanager.com/pdig/ and select the 'Submissions Needing Revision' folder to locate your manuscript file.

Please include the following items when submitting your revised manuscript:
* A rebuttal letter that responds to each point raised by the editor and reviewer(s). You should upload this letter as a separate file labeled 'Response to Reviewers'.
* A marked-up copy of your manuscript that highlights changes made to the original version. You should upload this as a separate file labeled 'Revised Manuscript with Track Changes'.
* An unmarked version of your revised paper without tracked changes. You should upload this as a separate file labeled 'Manuscript'.
If you would like to make changes to your financial disclosure, please include your updated statement in your cover letter. Guidelines for resubmitting your figure files are available below the reviewer comments at the end of this letter.

We look forward to receiving your revised manuscript.

Kind regards,

Iain Buchan, MD FFPH FACMI
Guest Editor
PLOS Digital Health

Journal Requirements:

1. Please amend your detailed Financial Disclosure statement. This is published with the article. It must therefore be completed in full sentences and contain the exact wording you wish to be published.

a. State what role the funders took in the study. If the funders had no role in your study, please state: "The funders had no role in study design, data collection and analysis, decision to publish, or preparation of the manuscript."
b. If any authors received a salary from any of your funders, please state which authors and which funders.

2. We ask that a manuscript source file is provided at Revision. Please upload your

manuscript file as a .doc, .docx, .rtf or .tex.

Please review your reference list to ensure that it is complete and correct. If you have cited papers that have been retracted, please include the rationale for doing so in the manuscript text, or remove these references and replace them with relevant current references. Any changes to the reference list should be mentioned in the rebuttal letter that accompanies your revised manuscript. If you need to cite a retracted article, indicate the article's retracted status in the References list and also include a citation and full reference for the retraction notice.

Additional Editor Comments (if provided):

Thank you for this well-reasoned paper on pain-mood endophenotype exploration, with methodology relevant to wider mental-physical health comorbidity research.

Please provide the clarifications the reviewers request, mostly in the supplementary material.

Please make your code available in a public repository, and encourage the data-controller to make suitable synthetic data available for easier knowledge-transfer of your methods/code.

Align your clarification of model selection with the code you publish, linked to from the supplementary material.

Clarify the cohort definition/characterisation with more information about https://www.cloudywithachanceofpain.com in the supplement.

Optionally, you may wish to elaborate on the use of (variable) longitudinal observation designs to distinguish patterns of resilience to mood/pain worsening from patterns of susceptibility, with potentially earlier tipping points and different sequencing.

[Note: HTML markup is below. Please do not edit.]

Reviewers' comments:

Reviewer's Responses to Questions

Comments to the Author

1. Does this manuscript meet PLOS Digital Health’s publication criteria? Is the manuscript technically sound, and do the data support the conclusions? The manuscript must describe methodologically and ethically rigorous research with conclusions that are appropriately drawn based on the data presented.

Reviewer #1: Yes

Reviewer #2: Yes

----

2. Has the statistical analysis been performed appropriately and rigorously?

Reviewer #1: Yes

Reviewer #2: Yes

----

3. Have the authors made all data underlying the findings in their manuscript fully available (please refer to the Data Availability Statement at the start of the manuscript PDF file)?

The PLOS Data policy requires authors to make all data underlying the findings described in their manuscript fully available without restriction, with rare exception. The data should be provided as part of the manuscript or its supporting information, or deposited to a public repository. For example, in addition to summary statistics, the data points behind means, medians and variance measures should be available. If there are restrictions on publicly sharing data—e.g. participant privacy or use of data from a third party—those must be specified.

Reviewer #1: No

Reviewer #2: No

----

4. Is the manuscript presented in an intelligible fashion and written in standard English?

PLOS Digital Health does not copyedit accepted manuscripts, so the language in submitted articles must be clear, correct, and unambiguous. Any typographical or grammatical errors should be corrected at revision, so please note any specific errors here.

Reviewer #1: Yes

Reviewer #2: Yes

----

5. Review Comments to the Author

Please use the space provided to explain your answers to the questions above. You may also include additional comments for the author, including concerns about dual publication, research ethics, or publication ethics. (Please upload your review as an attachment if it exceeds 20,000 characters)

Reviewer #1: Thank you for the opportunity to review this paper, which I enjoyed and found interesting and informative.

With specific reference to the PLOS Reviewer Guidelines

** What are the main claims of the paper and how significant are they for the discipline?

The authors use an existing, longitudinal mHealth data set consisting of > 9000 participants who provide daily, paired (mood,pain) ratings. The authors dichotomise pain and mood into "low" and "high" states to simplify the data to enable an EM fit for mixture Markov models such that for each person, they reside in one of four possible states and can transition to any of these four at the next measurement.

The authors show how they arrive at a k=4 cluster Markov chain model and provide post-hoc analyses of the distributions of pain conditions within/between the discovered clusters.

Overall, my impression is that this is novel work and methodologically sound (within the confines of my experience with these kinds of modelling techniques). The results are interesting and the limitations discussed appropriately. My interpretation of this paper is that the authors provide a novel method for analysing mHealth data in the context of multi-morbidity in pain syndromes (rather than, say, providing definitive population level analyses with clinical significance).

These methods would be applicable and have utility for researchers in other conditions/disorders where understanding paired associations over time would provide insights. For example, as the authors show, chronic headache and fibromyalgia (and to some extent, spondyloarthropathy) have higher log odds of being present in Cluster 2 participants and this represents an insight that mood/affective state modulates experiences of pain (or vice versa) in these conditions in a way that separates them from participants in other clusters.

I have only minor suggestions, outlined below, which I think would benefit the paper.

** Are these claims novel? If not, please specify papers that weaken the claims of originality of this one. If a similar paper was recently published, the current manuscript may still be eligible for publication under our complementary research policy?

The research is novel.

** Are the claims properly placed in the context of the previous literature? Have the authors treated the literature fairly?

Yes.

** Do the data and analyses fully support the claims? If not, what other evidence is required?

Yes.

** Would additional work improve the paper? How much better would the paper be if this work were performed and how difficult would it be to do this work?

I would suggest the following minor amendments

1) the authors don't provide detail on whether or not the participants were self-selecting and self-reporting diagnoses. This would help situate the paper for clinically-focused readers who might ask whether or not the participants were a clinical population (i.e. any diagnoses of mood disorders and/or confirmed diagnoses of pain syndromes / musculo-skeletal / rheumatoid disorders). The introduction helpfully describes the pain <-> mood bidirectional association, so it would be worth highlighting if this is a "clinical population" or a group of people self-selecting to trial monitoring their mood <-> pain symptoms. Alternatively, in the Limitations sections, the authors could clarify if this can be considered a clinical population or not. and what is known about participants' diagnoses.

2) I struggled to understand how the "intervention" (which appears to simulate changes in individual cluster's transition probability matrices) simulations were implemented -- for example, after fitting the k = 4 model, from my reading, it seems the authors then

set the beta_P and beta_M values (in addition to the transition probability matrix contraints -- constants 0.8 and 0.2 -- to impose an asymmetric transition probability BH and BL from BG and BL) so that beta_M < 0.15 and beta_P < 0.2. If the intention is to show that the model could allow one to simulate intervention, then I would either expand on this in the main text, or if this is less important, I would move to supplementary information -- as it stands, I couldn't find an a priori reason why this simulation was included in the main manuscript (especially given they chose the constants/penalties and beta values themselves). I think I understand the what the authors are attempting to show, but my overall impression is that this is somewhat arbitrary and I'm not entirely clear that it adds much to the results presented.

3) Model selection: The authors argue for a qualitative "elbow" in the negative log-likelihood implying a solution with clusters k = 4 (Figure S4, section S.1.4) and that "4-component mixture had certain natural interpretations". While this is reasonable, I wondered why the authors did not select clusters using a method that explicitly penalises for model complexity (i.e. the number of parameters) such as BIC ? If the data supports a k = 4 cluster model, one would expect the resulting penalised neg log lik to be parabolic, with a minimum at 4 -- if this is the best compromise between under/over-fitting the number of clusters ? Perhaps the authors could comment on this in Suppl Info.

** If a protocol is provided, for example for a randomized controlled trial, are there any important deviations from it? If so, have the authors explained adequately why the deviations occurred?

Not relevant to this work.

** PLOS Digital Health encourages authors to publish detailed protocols and algorithms as supporting information online. Do any particular methods used in the manuscript warrant such treatment?

I defer to the editor on this issue, but my view is that for methods such as those presented, the authors should place their code in a public repository so others can inspect the finer-grained implementation of the methods. No libraries/implementation language is mentioned in the text, which suggests the authors implemented the methods themselves, highlighting the need for transparent code. The data is highlighted as being owned by a party that is not the authors, so I would encourage them to provide some minimal, simulated example data so others can explore their code/implementation without needing to locate similar data.

** If the paper is considered unsuitable for publication in its present form, does the study itself show sufficient potential that the authors should be encouraged to resubmit a revised version?

Not relevant

** Are original data deposited in appropriate repositories and accession/version numbers provided for genes, proteins, mutants, diseases, etc.?

See above : the authors indicate they are unable to provide the data and on the cited project website (https://www.cloudywithachanceofpain.com/blog), there is no data sharing statement that I could locate.

** Does the study conform to any relevant guidelines such as CONSORT, MIAME, QUORUM, STROBE, and the Fort Lauderdale agreement?

Not relevant.

** Are details of the methodology sufficient to allow the experiments to be reproduced?

Partially, see above.

** Is any software created by the authors freely available?

See above.

** Is the manuscript well organized and written clearly enough to be accessible to non-specialists?

Yes.

Reviewer #2: This is a nicely written study aiming to identify patterns of pain and mood reporting in a large electronic health dataset. They show that although pain and mood are known to be associated, there are groups of people who have different relationships between pain and mood, showing 4 distinct categories. The approach is appropriate and interesting, and the findings may have value in future targeting of personalised treatments for pain and low mood.

I only have a few minor comments and requests for clarification.

1. For how many days were participants expected to respond on the App? What happened if a person missed one day but then continued to fill in the app?
2. How were the odds ratios in tables S2 and S3 calculated? Something like a multinomial model? Can this be clarified in the methods section please?
3. The choice has been made to identify 4 clusters. However, in Figure S4, more clusters provide better log-likelihood. The justification is that the reduction is not much once you have more than 4 clusters. But this is a bit arbitrary. It would be good to discuss this a little more and describe what impact this might have. How strongly should we believe that there are four patterns of behaviour for example? In say the 8 or 10 cluster model, do you get lots of similar clusters, or do you find additional patterns to the ones you have identified? The abstract says that you have discovered four distinct patterns – how reliable is this?
4. What impact do you think reducing the states to 2 per variable has? It's certainly simpler, but do you think it loses useful information on trajectory?
5. How reasonable is the Markov assumption in equation 3? I wonder the next state being only dependent on the previous state (and not how it got there) is unlikely to hold – especially in the 25-state model. I suspect this may be less of an issue in the collapsed model presented here with only 2 categories for each of pain and mood. But in the 5 original categories might it not be the case that someone in state 3 was more likely to go to state 4 if they had shown a constant rise from state 1 through to state three than if they had just arrived in state 3 from state 4?
6. The clustering of Markov processes described in this paper is a neat idea. Setting this problem up as a set of states and looking at transitions between them is certainly a natural way to think. I do find clustering of transition matrices a little hard to conceptualise though. There are other ways of doing longitudinal clustering that might work in this setting. For example, latent class trajectory models (Proust-Lima, C., Philipps, V. and Liquet, B., 2015. Estimation of extended mixed models using latent classes and latent processes: the R package lcmm. arXiv preprint arXiv:1503.00890.) or multivariate mixture models (Komárek, A. and Komárková, L., 2013. Clustering for multivariate continuous and discrete longitudinal data. The Annals of Applied Statistics, 7(1), pp.177-200.). I think these would be suitable for your problem (specially with binary pain and mood scores). I'm not proposing redoing any analysis, but I think it would be useful to describe the relative methods of your approach compared to these other examples of approaches to doing essentially the same thing.

----

6. PLOS authors have the option to publish the peer review history of their article (what does this mean?). If published, this will include your full peer review and any attached files.

Do you want your identity to be public for this peer review? If you choose “no”, your identity will remain anonymous but your review may still be made public.

For information about this choice, including consent withdrawal, please see our Privacy Policy.

Reviewer #1: No

Reviewer #2: No

[NOTE: If reviewer comments were submitted as an attachment file, they will be attached to this email and accessible via the submission site. Please log into your account, locate the manuscript record, and check for the action link "View Attachments". If this link does not appear, there are no attachment files.]

While revising your submission, please upload your figure files to the Preflight Analysis and Conversion Engine (PACE) digital diagnostic tool, https://pacev2.apexcovantage.com/. PACE helps ensure that figures meet PLOS requirements. To use PACE, you must first register as a user. Registration is free. Then, login and navigate to the UPLOAD tab, where you will find detailed instructions on how to use the tool. If you encounter any issues or have any questions when using PACE, please email PLOS at figures@plos.org. Please note that Supporting Information files do not need this step.

In compliance with data protection regulations, you may request that we remove your personal registration details at any time. (Use the following URL: https://www.editorialmanager.com/pdig/login.asp?a=r). Please contact the publication office if you have any questions.
